# Ground Layer Plant Species Turnover and Beta Diversity in Southern-European Old-Growth Forests

**DOI:** 10.1371/journal.pone.0095244

**Published:** 2014-04-18

**Authors:** Francesco Maria Sabatini, Sabina Burrascano, Hanna Tuomisto, Carlo Blasi

**Affiliations:** 1 Department of Environmental Biology, Sapienza, University of Rome, Rome, Italy; 2 Department of Biology, University of Turku, Turku, Finland; DOE Pacific Northwest National Laboratory, United States of America

## Abstract

Different assembly processes may simultaneously affect local-scale variation of species composition in temperate old-growth forests. Ground layer species diversity reflects chance colonization and persistence of low-dispersal species, as well as fine-scale environmental heterogeneity. The latter depends on both purely abiotic factors, such as soil properties and topography, and factors primarily determined by overstorey structure, such as light availability. Understanding the degree to which plant diversity in old-growth forests is associated with structural heterogeneity and/or to dispersal limitation will help assessing the effectiveness of silvicultural practices that recreate old-growth patterns and structures for the conservation or restoration of plant diversity. We used a nested sampling design to assess fine-scale species turnover, i.e. the proportion of species composition that changes among sampling units, across 11 beech-dominated old-growth forests in Southern Europe. For each stand, we also measured a wide range of environmental and structural variables that might explain ground layer species turnover. Our aim was to quantify the relative importance of dispersal limitation in comparison to that of stand structural heterogeneity while controlling for other sources of environmental heterogeneity. For this purpose, we used multiple regression on distance matrices at the within-stand extent, and mixed effect models at the extent of the whole dataset. Species turnover was best predicted by structural and environmental heterogeneity, especially by differences in light availability and in topsoil nutrient concentration and texture. Spatial distances were significant only in four out of eleven stands with a relatively low explanatory power. This suggests that structural heterogeneity is a more important driver of local-scale ground layer species turnover than dispersal limitation in southern European old-growth beech forests.

## Introduction

The composition of plant species assemblages varies in space and time as a result of the complex interplay among several structuring factors [1]. Spatial distribution of plant species depends on different mechanisms related to their tolerances to environmental factors, intra- and inter-specific interactions (e.g. dispersal, competition, herbivory, pathogens) and random variation. Given the theoretical and practical relevance of understanding these drivers, they have received much attention in recent years [2]–[7].

The relative importance of the mechanisms that generate floristic turnover can differ widely among systems as a consequence of habitat type, biogeographical context, plant life-history traits and other local contingencies [5], [8], [9]. The spatial scale of the study and the length of the relevant ecological gradients may also have an effect. When the sampled ecological gradient is long, i.e. when a wide range of environmental conditions are considered, resource-driven processes are expected to have a stronger influence on the community than when the gradient is truncated, since focusing on small geographic extents and relatively homogeneous habitats can lead to higher noise-to-signal ratios [10]–[13]. At local scales, therefore, floristic patterns may be expected to mostly reflect the effects of dispersal limitation and random survival [7], [14]. This complexity has fostered a long-standing debate on the degree to which community assembly is a result of niche vs. neutral processes, which was revived after the publication of Hubbell's book on the neutral theory of biodiversity [15]. Most studies addressing the issue since then have found both neutral and niche processes to operate simultaneously [2], [8], [9], [14], [16].

In temperate forests, niche and neutral processes may concurrently control local-scale variation of ground layer species composition, although their relative roles may vary with successional stage. As late-successional stands develop, structural heterogeneity (vertical and horizontal) and the number and variety of ecological niches (e.g. microhabitat) increase [17]–[19]. The persistence of relatively stable ecological conditions for a long time (ecological continuity), may allow for the emergence of patterns due to the colonization and persistence of low-dispersal species dependent on very old, undisturbed forests [20]. Both the accumulation of microhabitats and the colonization of forest interior species depend on time since last disturbance, although it is not clear what their relative roles are in determining local-scale variation of ground layer composition.

An integrated approach is necessary to assess the individual contributions of structural heterogeneity and dispersal limitation to explaining species turnover, because most ecologically relevant variables are spatially autocorrelated [5], [7]. Understorey plants can be affected by factors determined by overstorey structure, such as light availability, but also by purely abiotic factors, such as soil properties and topography. In principle, after accounting for all sources of environmental heterogeneity, the residual correlation between geographical distances and species turnover can be considered a proxy of spatially autocorrelated biological processes such as dispersal limitation or other biotic interactions [21].

The study of the mechanisms that cause biological diversity to accumulate and be maintained in natural ecosystems at different spatial scales and under different conditions is an essential foundation of management and conservation strategies implemented at the local, landscape and regional levels [3], [22], [23]. Old-growth forests represent a reference point for evaluating human impacts on other forest ecosystems and for improving current sustainable forest management practices. These forests have avoided severe disturbance for centuries, and species distribution can therefore be expected to reflect environmental gradients and demographic dynamics more clearly than in managed forests, where anthropogenic contingencies (such as land-use history and forest management) dominate [4], [24]–[26]. Old-growth remnants, thus represent an ideal situation for investigating the dynamics and patterns of ground layer vegetation under natural conditions, although their relative scarcity in several biogeographic regions is a challenge for unravelling the drivers of community assembly. This is particularly true for Europe, where forests have been impacted by man for millennia and are likely to be, on average, more managed than forests in most parts of the world [24]. Understanding the degree to which plant diversity in old-growth forests is associated with structural heterogeneity and/or to dispersal limitation will help assessing the effectiveness of silvicultural practices that recreate old-growth patterns and structures for the conservation or restoration of plant diversity.

Here, we used a nested sampling design to study beta diversity and species turnover (i.e. the proportion of species composition that changes among sampling units (*sensu* Tuomisto [36], [37]) across 11 of the best preserved beech forest stands with old-growth characteristics in Southern Europe. We primarily focused on ground layer flora because it hosts the vast majority of forest biodiversity [27], and plays an important role in the structure and functioning of forest ecosystems due to its influence on nutrient fluxes, tree regeneration, successional patterns and light regime [28], [29].

We hypothesized that after accounting for the intrinsic heterogeneity due to abiotic factors, most of the variation in ground layer species turnover is related to stand structural heterogeneity. However, we also expected to observe a significant effect of dispersal limitation [7], [14], [23]. Finally, we hypothesized that the relative importance of different environmental and structural variables would vary across the stands according to their degree of fine-scale heterogeneity [10], [11].

## Materials and Methods

### Study Sites

We selected 11 forests identified in the literature as being old-growth or having old-growth characteristics. These stands encompass four different countries in Southern Europe (Italy, Spain, Bosnia-Herzegovina and Montenegro) and are dominated by European beech (*Fagus sylvatica*); codominant species vary across stands and encompass silver fir (*Abies alba*), Turkish oak (*Quercus cerris*), or sessile oak (*Quercus petraea*). All study sites belong to the temperate oceanic bioclimate [30]. Eight out of 11 stands grow on sedimentary bedrock (limestone, marlstone, sandstone), two on eruptive rock, and one on metamorphic rock (Table 1). All necessary permits were obtained for the described study, which complied with all relevant regulations. Depending on the forest stand, the permits were issued by the competent National Parks' authorities, Italian State Forestry Corps, ARIF Puglia or by the Consejería de Agroganadería y Recursos Autóctonos, Gobierno del Principado de Asturias.

**Table 1:** Location and main features of the 11 stands with old-growth characteristics included in this study.

Forest Name	Location	Nation	Altitude	Average mean Temp. (°C)	Average Annual Prec. (mm)	Year since last human intervention	Dominant Species	Bedrock	Phytosociological type	Ref.
Abeti Soprani	Central Apennines	I	1250–1450	8.4	1124	30	AA, FS	Marly Limestone	*Pulmonario apenninae- Abietetum albae*	[31], [61]
Biogradska Gora	Biogradska Gora NP	Mne	1300–1350	2–6	1750–2000	140	FS, AA	Eruptive rocks	*Abieti-Fagetum*	[32], [62]
Val Cervara	Abruzzo, Lazio, Molise NP	I	1730–1830	7.2	1211	no ref	FS	Limestone	*Cardamino kitaibelii- Fagetum sylvaticae*	[18], [31], [61], [63], [64]
Monte Cimino	Central Italy	I	925–1053	14.3	1300	61	FS	Trachyte	*Allio pendulini- Fagetum sylvaticae*	[31], [61]
Collemeluccio	Central Apennines	I	900–1000	9.2	960	50	AA, FS, QC	Marly limestone	*Pulmonario apenninae- Abietetum albae*	[31], [61]
Fonte Novello	Gran Sasso, Monti della Laga NP	I	1340	10	1071	310	FS	Sandy marlstone	*Cardamino kitaibelii- Fagetum sylvaticae*	[31], [61], [65]
Gargano Pavari	Gargano NP	I	720–800	11.6	1041	56	FS	Limestone	*Anemono apenninae- Fagetum sylvaticae*	[31], [61]
Monte di Mezzo	Central Apennines	I	950–1150	8.6	1022	55	FS, QC	Marly Limestone	*Anemono apenninae- Fagetum sylvaticae*	[31], [61]
Muniellos	Asturias	E	1200–1250	13	1980	70	FS, QP	Quartzite, sandstone	*Blechno spicant-Fagetum sylvaticae*	[66], [67]
Perucica	Sutjeska NP	BiH	1450–1500	8.6	1363	no ref	AA, FS, PA	Limestone, Sandstone	*Piceo-Abieti-Fagetum*	[32], [56], [68]
Sasso Fratino	Foreste Casentinesi NP	I	950–1050	9	1689	76	FS, AA	Sandstone, Marlstone	*Cardamino heptaphyllae- Fagetum sylvaticae; Aceri platanoidis-Fagetum sylvaticae*	[31], [61], [69], [70]

Nations – I: Italy, E: Spain, BiH: Bosnia and Herzegovina, Mne: Montenegro. Dominant species – AA: *Abies alba*, FS: *Fagus sylvatica*, QC: *Quercus cerris*, QP: *Quercus petraea*, PA: *Picea abies*.

### Sampling design

We used a nested sampling design. In each forest stand, we set a network of 25 sampling quadrats regularly distributed in a 1-ha square macroplot. Quadrats were 5 m×5 m and were located at the centres of a regular grid consisting of 20 m×20 m cells. For each quadrat we sampled vascular plant species composition, forest structure (live and deadwood) and environmental variables (topography, soil, light). The macroplots were positioned in the field in the same locations where previous research projects had investigated structural features of the stands [31], [32].

The vegetation was divided into three layers: tree (height >3 m), shrub (1.3< height ≤3 m) and ground layer (height ≤1.3 m). We estimated plant cover using an ordinal cover class scale with class limits 0.5%, 1%, 2%, 5%, 10%, 15%, 20%, and thereafter every 10% up to 100% (each class includes its upper limit). Each plant species was assigned a cover value in each layer separately through visual estimation, and in addition we recorded the total cover of the tree and shrub layer.

For all trees with dbh >2.5 cm situated inside the quadrats, we recorded the species, diameter at breast height (dbh), and vitality class (1 = vigorous; 2 = living with dead parts; 3 = standing dead; 4 = broken above 1.3 m). This information was used to calculate total basal area of live trees within the quadrats. We also used a wedge prism to estimate the basal area per hectare on the basis of a variable radius plot centred on each quadrat. Hereafter, we will refer to the former measure as ‘quadrat basal area’ and to the latter as ‘prism basal area’. For each quadrat we also measured the position, species and diameter of the four large live trees with dbh>40 cm closest to the quadrat centre. These data were used to calculate three indices of structural heterogeneity [33]. The first describes spatial distribution of large live trees (*Uniform Angle Index*, also known as Winkelmass Index), the second species clustering (*Species Mingling Index*) and the third diameter heterogeneity (*Modified Dbh Dominance Index* or DBHDM). The average of four readings of a hemispherical densiometer taken in the four cardinal directions was used to estimate the openness of the overstorey canopy cover (‘canopy openness’ hereafter).

Diffuse light transmittance of photosynthetically active radiation (µmol/m^2^•sec, hereafter ‘PAR’) at 1 m height was measured using a *Licor Line Quantum Sensor - Li-191* under uniform sky conditions at three different times of day: morning (10–11 am), noon (12.30–1.30 pm) and afternoon (3–4 pm). In the stand Muniellos, all measurements were taken an hour later. Two measurements were taken within each quadrat, 1.5 m north and 1.5 m south of the quadrat centre, and their average was used in the analyses. An overall average of all six measurements per quadrat was also calculated.

For every deadwood piece of at least 10 cm length and 5 cm minimum diameter occurring in the quadrat, we measured the two end-diameters and length, identified the species when possible and assigned the piece to one of five decay classes [34]. Each quadrat was characterised by the total number of such deadwood pieces, their total volume (assuming a truncated cone shape for each piece), the number of different decay classes present, and the most advanced state of decay present.

The following micro-topographical variables were recorded for each quadrat: slope, aspect, topographical position (ridge top, upper slope, mid slope, lower slope, valley, flat area), and percentage cover of outcropping rocks and stones. Data on slope and aspect, together with stand latitude, were used to calculate above-canopy potential annual direct incident solar radiation (MJ/cm^2^•yr) [35].

A topsoil sample (a single core of the top 20 cm) was collected for each quadrat, and analysed in the laboratory for granulometry (Bouyoucos's densimetric method), pH (in distilled water), carbon concentration (Walkley and Black's method) and total nitrogen concentration (Kjeldahl's method). In the field we estimated litter depth and the percentage of ground covered by litter. We also measured soil volumetric water content (VWC, the ratio of water volume to soil volume) in each quadrat as the average of five measurements done with a soil moisture meter *Field Scout - TDR 100*.

For each stand, we noted the occurrence of different kinds of disturbance related to timber harvest (e.g. occurrence of man-made tree stumps), wild boar rooting, trampling and surface water soil erosion. For each kind of disturbance we subjectively assigned an ordinal value ranging from 0–3 (0 = none, 1 = low, 2 = moderate, 3 = severe). A synthetic index of disturbance was then calculated as the sum of the four individual indices.

Sampling was performed in late spring-early summer in years 2011–2012. Stands located at low altitude or with higher average temperature were sampled earlier during the growing season in order to sample all the stands in a comparable phenological stage.

### Beta diversity

A wide range of different phenomena, including various kinds of heterogeneity, differentiation and rate of change, with or without reference to external explanatory factors are often referred to the term beta diversity [36]–[38]. To avoid confusion, here we use the term beta diversity only to refer to the effective number of compositionally distinct sampling units (which in our case corresponds to 5 m×5 m quadrats). This equals true beta diversity as defined by Tuomisto [36]–[39] on the basis of the foundation laid by Hill [40] and Jost [41], [42]. When referring to the proportion of species composition that changes among sampling units, we use the term species turnover instead [37], [38].

For each stand we calculated species diversity as follows [36], [40]:
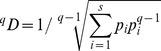
(1)where *p_i_* is the proportional abundance of species *i*, S is the total number of species, and *q* is the order of the diversity. We calculated true diversity both with q = 0 and with q = 2. When q = 0, species abundances cancel out from the equation, so ^0^D obtains the same numeric value as species richness. When q>1, abundant species are given more weight than implied by their proportional abundances, and at q = 2 the denominator of eq. (1) equals the original Simpson diversity index [41], [42].

Species diversity observed at a particular spatial extent (in our case, within the 1-ha plot) can be thought to result from two independent factors observable at a smaller spatial grain, namely average species density within sampling units (the 5 m×5 m quadrats) and compositional heterogeneity among the sampling units. To quantify the relative roles of these, we followed Tuomisto [37] to perform a multiplicative partitioning of the total observed species diversity in each stand (gamma diversity): ^q^D_γ_  = ^q^D_α_ × ^q^D_β_. We developed for this purpose an *ad hoc* R script that is provided in Appendix S1.

### Multiple regression on dissimilarity matrices

There has been some controversy on whether beta diversity is better modelled with the raw-data or the distance approach [21], [43], which partly stems from different definitions of beta diversity. Tuomisto and Ruokolainen [21] argued that both approaches can be justified in different situations, and that the choice of the statistical method depends on the ecological question of interest.

Here we were primarily interested in the relative contributions of environmental differences and dispersal limitation in explaining species turnover, which is a question spelled out in terms of distances (a level-3 question according to Tuomisto and Ruokolainen [21]). Therefore, we adopted a distance-based variation partitioning approach, i.e. we partitioned the variation of dissimilarity matrices, that are based on species abundance data, into fractions uniquely or jointly explained by a number of explanatory dissimilarity matrices [2], [44], [45].

Species turnover was calculated for sampling unit pairs with a dissimilarity measure that is a monotonic transformation of true beta diversity and can be thought of as a generalization of the Jaccard similarity index to abundance data [36], [41]:
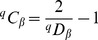
(2)


The complement of this index is a dissimilarity measure linearly related to proportional species turnover (*sensu* Tuomisto [37]). We used this measure with q = 2 to build a quadrat-to-quadrat dissimilarity matrix for each stand separately, based on ground layer species composition. Corresponding dissimilarity matrices were built based on each structural and environmental variable separately, using either the Euclidean distance (for quantitative variables, after standardization) or Gower dissimilarity (for ordinal, nominal and binary variables). Finally, we built a matrix of geographical distances between the centre-points of the quadrats.

Explanatory variables were organized into three sets: 1) forest structure, 2) abiotic environmental factors and 3) geographical distances (Table 2). Mean values and standard deviation of the original environmental variables in each stand are shown in table S1. Forest floor PAR was included among the structural variables, because it is mostly determined by canopy features. Set 3 contained a single distance matrix, that of linear spatial distances among quadrats. To check if species turnover is related to geographical distances in a non-linear manner, we ran the analyses also with distance matrices that had been log-transformed or rank-transformed, but the results were very similar and are not shown.

**Table 2:** Structural and environmental variables used to calculate explanatory dissimilarity matrices; sets and subsets in which the variables were included; type of variable (nominal: N, ordinal: O, quantitative: Q); and unit of measurement (range for ordinal variables).

Set	Subset	Variable used to calculate dissimilarity	Variable type	u.m.
1.Structure	1a.Composition	Tree richness	Q	n species
	1b. Live structure	Tree cover	Q	%
		Shrub cover	Q	%
		Developmental phase	O	1–5
		Basal Area (Prism)	Q	m^2^/ha
		Stem density	Q	n/25 m^2^
		Basal area (quadrat)	Q	m^2^/25 m^2^
		Canopy openness	Q	%
		Uniform Angle Index	Q	0–1
		Sp. Mingling Index	Q	0–1
		DBHDM Index	Q	0–1
		Distance closest large live tree	Q	M
	1c. Deadwood	Deadwood volume	Q	m^3^/25 m^2^
		Deadwood density	Q	n/25 m^2^
		Max decay class	O	0–5
		Num. decay classes	Q	0–5
	1d. Ground layer transmitted Light	Morning PAR	Q	µmol/m^2^•sec
		Noon PAR	Q	µmol/m^2^•sec
		Afternoon PAR	Q	µmol/m^2^•sec
		Average PAR	Q	µmol/m^2^•sec
2. Abiotic Environment	2a. Topography	Slope	Q	°
		Folded aspect	Q	°
		Pot. solar irradiation	Q	ln(MJ/cm^2^•yr)
		Topographic position	N	
		Slope position	N	
		Rock coverage	Q	%
		Stone coverage	Q	%
	2b. Soil	Soil pH	Q	
		Soil Organic Matter	Q	% (g/g)
		Soil tot. N	Q	% (g/g)
		C/N ratio	Q	%
		Soil stone content	O	0–3
		Coarse Sand %	Q	% (g/g)
		Medium Sand %	Q	% (g/g)
		Fine Sand %	Q	% (g/g)
		Silt %	Q	% (g/g)
		Clay %	Q	% (g/g)
		Texture	N	
		Soil water content	Q	%
		Litter cover	Q	%
		Litter depth	Q	cm
	2c. Disturbance	Disturbance-Trampling	O	0–3
		Disturbance-Rooting	O	0–3
		Disturbance-Timber harvest	O	0–3
		Disturbance-Water Erosion	O	0–3
3. Space	3a. Geographical location	X and Y coordinates	Q	m

To quantify the variation explained by each set of explanatory variables in each stand, we first performed a preliminary selection of dissimilarity matrices that have a significant marginal effect on ground layer species turnover through simple Mantel tests (9,999 permutations). We then performed a separate Multiple Regression on Distance Matrices analysis (MRM [44], [46]) for each of the three sets of explanatory variables, starting with all the variables that were individually significant and removing the non-significant ones through backward elimination. All explanatory variables that remained in any of the three models were then used to quantify the total variation explained by a full MRM model. Finally, we followed the standard decomposition techniques used in variation partitioning to extract ‘unique’ and ‘shared’ fractions of variance explained by the three sets of variables [2], [45], [47].

For each quantitative environmental and structural variable, we finally tested whether the marginal effect of its dissimilarity matrix was correlated (Pearson's ρ) with the standard deviation of that variable in different stands.

### Multivariate dispersion around group centroids

Compositional heterogeneity can be quantified with the average dissimilarity of individual sampling units from their group centroid in a multivariate species space. This is known as multivariate dispersion [48]. When the same dissimilarity measure is used, multivariate dispersion is monotonically related to the mean of pairwise dissimilarities between sampling units [36]. We illustrated the dispersion of quadrats around their stand centroids in the space defined by the first two axes of a Principal Coordinates Analysis (PCoA) based on the quantitative Jaccard dissimilarity measure. Stand centroids were calculated as the median of the quadrats in the PCoA-derived species space [48]. We also passively projected stand-level environmental variables (Table 1) on the graph.

We used a linear mixed effect model to incorporate the across-stand variation into the estimation of the overall response of multivariate dispersion to different explanatory variables. Compared to MRM, this analysis uses random effects (i.e. stands) to separate between the within-stand and among-stand effects of variables. Pairwise dissimilarities cannot be used in a mixed effects model since they are not independent of each other. Although the distances to centroid are not completely independent either, non-independence is not a serious problem with reasonable sample sizes [48].

We included as fixed effects of the initial mixed effect model the multivariate dispersions based on compound dissimilarity matrix derived from eight subsets of explanatory variables, i.e. overstorey composition (subset 1a), live structure (1b), deadwood (1c), light availability (1d), topography (2a), soil (2b), disturbance (2c) as well as spatial distances (3, Table 2). Dissimilarities were mostly calculated using Gower dissimilarity, but for subset 1a we used the complement of the Jaccard index (presence-absence data) and for subset 3a we used the Euclidean distance. We also included in the fixed part of the initial full model the average stand altitude and climatic variables (annual average precipitation and temperature) to test whether these stand-level variables explained species turnover.

Model selection was performed following a standard protocol [49]. We started with a model containing all the explanatory variables; we then chose an appropriate random part comparing different models (e.g. random intercept or random intercept and slope) using REML (Restricted Maximum Likelihood) estimation and AICs. The fixed part of the model was selected through backward elimination of non-significant variables through likelihood ratio test and ML estimation. After model validation, two quadrats were eliminated as outliers from the model; both of them were characterized by extreme disturbance either due to wild boars or water erosion along a steep slope.

All statistical computations were performed in R 3.0.0 [50]. Multivariate dispersion from group centroid were calculated using ‘vegan’ package (version 2.0–7). Mixed effect models were computed in R using ‘nlme’ package (version 3.1–105).

## Results

### Ground layer diversity

A total of 238 species were found in the 11 stands (25 in the tree layer, 21 in the shrub layer and 231 in the ground layer), accounting for a total of 4,541 species•layer•quadrat observations. The stands differed both in species richness and in the evenness of the species abundances within them (Table 3). As a result, the stands ranked differently in species diversity when species abundances were weighted in different ways and all diversity components were lower with q = 2 (which gives more weight to abundant species) than when q = 0.

**Table 3:** Decomposition of true gamma diversity (total species diversity in a site) into true alpha diversity (mean species density per quadrat) and true beta diversity (effective number of compositionally distinct quadrats) in 11 old-growth beech forest stands in southern Europe.

	alpha	beta	gamma	alpha	beta	gamma
Abeti Soprani	19.04	3.26	62	9.17	2.63	24.13
Biogradska Gora	15.76	3.24	51	4.93	2.32	11.43
Valle Cervara	11.24	4.09	46	4.68	1.97	9.23
Monte Cimino	13.08	2.98	39	4.41	2.88	12.68
Collemeluccio	12.92	4.26	55	2.86	1.74	4.98
Fonte Novello	9.08	3.19	29	3.15	1.54	4.87
Gargano-Pavari	5.48	4.01	22	2.85	2.36	6.73
Monte di Mezzo	7.16	4.47	32	3.49	2.56	8.96
Muniellos	5.24	4.77	25	1.46	1.27	1.84
Perucica	15.96	2.32	37	3.45	1.24	4.28
Sassofratino	14.60	3.63	53	8.04	3.05	24.50

Diversities were quantified at q = 0 (which gives more weight to rare species) and q = 2 (which gives more weight to abundant species).

Beta diversity (^2^D_β_) was positively related both to alpha diversity (Spearman's *ρ* = 0.67, p = 0.023) and to gamma diversity (*ρ* = 0.80, p = 0.003). None of the diversity measures was related to the values of the available stand-level descriptors (Table 1) or the means of most quadrat-level descriptors (Table 2). The exception was average PAR, for which there was a negative relationship with beta diversity (*ρ* = −0.82, p = 0.002). In other words, sites with more light were compositionally less heterogeneous. Furthermore, heterogeneity in the structural and environmental variables (as quantified with their standard deviations) were only related to the diversity components in two cases. Alpha diversity was positively related to heterogeneity in soil water content (*ρ* = 0.62, p = 0.043), and beta diversity was positively correlated with heterogeneity in soil C/N ratio (*ρ* = 0.80, p = 0.003).

### Dependence of pairwise species turnover on forest structure, environmental variables and spatial distances

**Figure 1:**
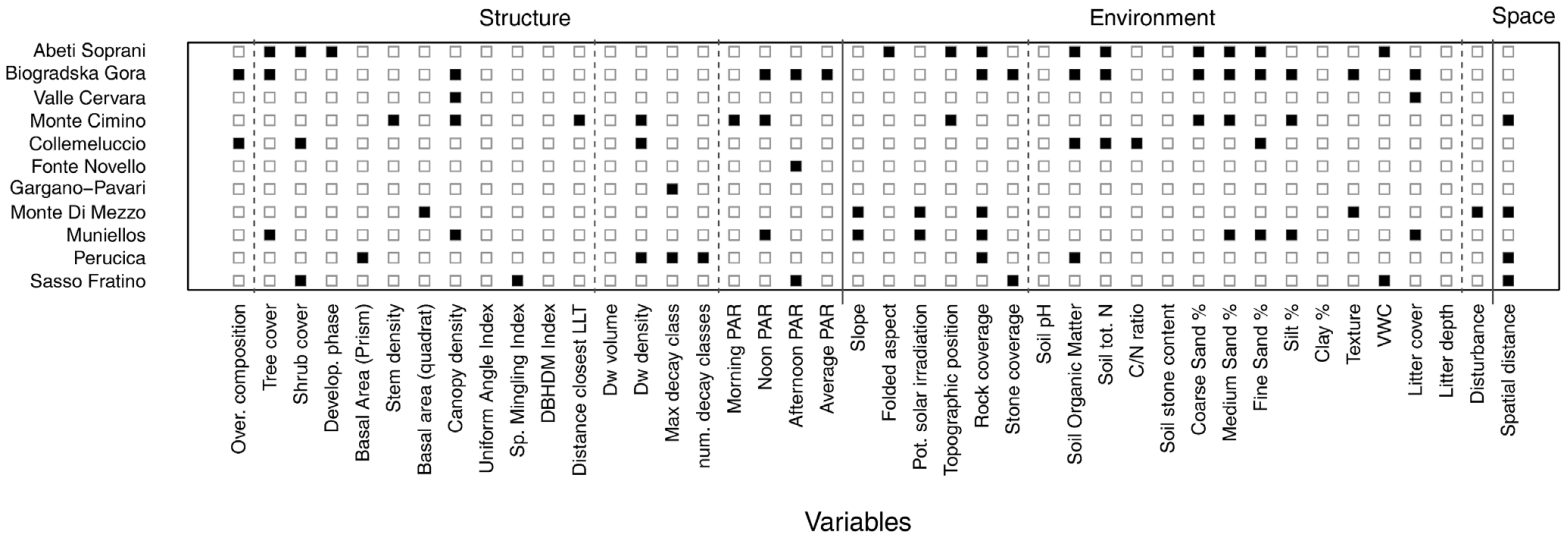
Significant Explanatory Variables. Variables that yield distance matrices with a significant (p<0.05) marginal effect (black quadrats) in mantel test (9,999 permutations) when modeling the ground layer plant species turnover in 11 old-growth beech forest stands in southern Europe.

The results of multiple regression on dissimilarity matrices differed greatly among stands both in terms of explanatory power and in the explanatory variables retained in the final model (Fig. 1). Variables directly or indirectly related to light availability (i.e. tree layer cover, canopy openness and ground layer PAR at different times of the day) were significant in seven of the 11 stands, and so were variables related to site topography (e.g. potential solar irradiation, topographic position, and rock coverage).

Other structural variables that significantly explained ground layer species turnover were mostly related to basal area and stem density (three stands) and to deadwood density and decay class (four stands). Species turnover in the tree layer explained species turnover in the ground layer only in the two stands with the highest tree species richness. The most important soil variables were related to texture, organic matter and total nitrogen concentration. Disturbance was significant only in the stand ‘Monte di Mezzo’. Finally, between-quadrats spatial distance was significant in four stands.

**Figure 2:**
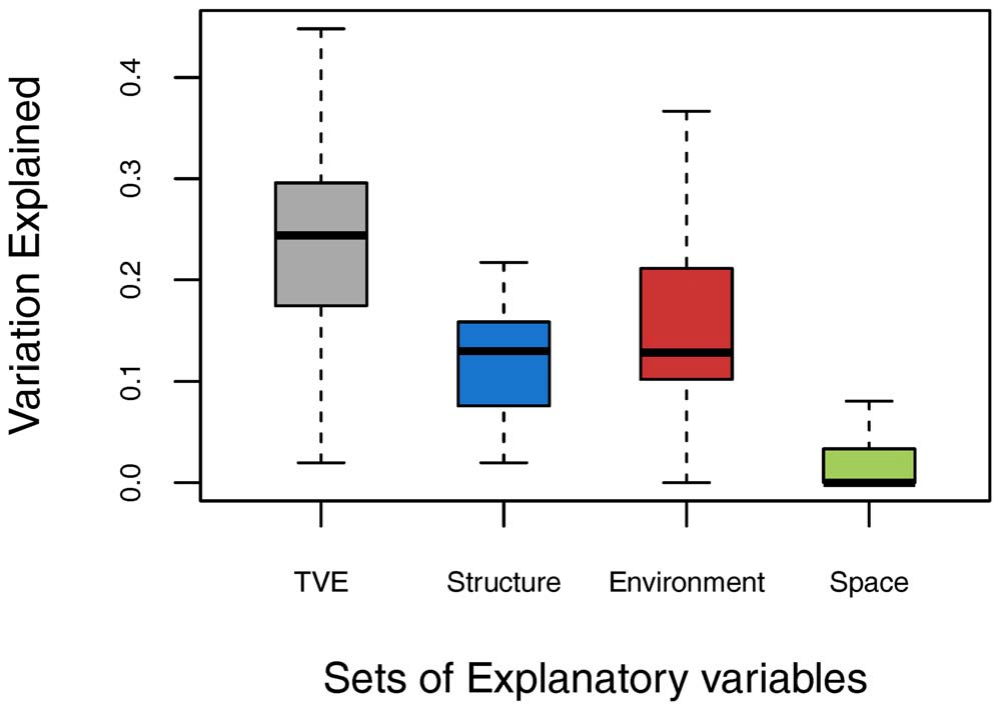
Variation Explained across stands. Boxplot showing the variability in how well ground layer species turnover was explained by multiple regression on distance matrices models (MRMs) in 11 old-growth beech stands in southern Europe. The first three boxplots correspond to models where explanatory variables are based on forest structural distances only, abiotic environmental distances only and spatial distances only. The last boxplot reports on the total variation explained (TVE) by the full models that include the explanatory variables of all three categories.

The total variance in species turnover explained by the MRM models ranged from less than 2.0% (in stand ‘Gargano-Pavari’) to 44.8% (in ‘Muniellos’) with a median of 24.4% (Fig.2). Species turnover mainly responded to between-quadrats structural dissimilarities, which contributed to between 2% (in ‘Gargano-Pavari’) and 21.7% (in ‘Biogradska Gora’) of the explained variance, with a median of 13.0% (sum of the unique and shared fractions involving structural dissimilarities). The abiotic environmental dissimilarities contributed to between 0% (in ‘Fonte Novello’ and ‘Gargano-Pavari’) and 36.7% (in ‘Muniellos’) of the explained variance, with a median of 12.8% (Fig. 2). Between-quadrat spatial distances were significant only in four of the eleven stands, and contributed to between 0% and 8.1% (median  = 0%) of the explained variance. The high total variance explained reported for three stands (‘Muniellos’, ‘Biogradska Gora’ and ‘Abeti Soprani’) was mostly related to their environmental heterogeneity (explaining respectively 36.7%, 30% and 25.1% of species turnover variation, Table 4), especially in soil and topographical features (Table S1 and S2).

**Table 4:** Results of multiple regression on distance matrices analyses (MRMs) run on ground layer species turnover in 11 old-growth beech forest stands in southern Europe.

		Percentage of variation explained due to ‘pure’ effects and shared fractions						
	Full model	Forest structure	Abiotic environment	Spatial	Str:Env| Spa	Str:Spa| Env	Env:Spa| Str	Str:Env:Spa
Abeti Soprani	30.3	5.2	17.3	-	7.8	-	-	-
Biogradska Gora	37.0	7.0	15.3	-	14.7	-	-	-
Valle Cervara	15.6	5.9	7.8	-	2.0	-	-	-
Monte Cimino	19.3	7.0	4.4	1.0	5.4	0.6	0.6	0.2
Collemeluccio	24.4	11.6	9.7	-	3.1	-	-	-
Fonte Novello	7.3	7.3	-	-	-	-	-	-
Gargano-Pavari	2.0	2.0	-	-	-	-	-	-
Monte di Mezzo	28.9	10.0	20.7	10.1	−7.7	−8.4	−5.0	9.1
Muniellos	44.8	8.1	27.0	-	9.6	-	-	-
Perucica	28.5	9.2	8.8	3.5	2.4	1.8	3.3	-0.6
Sassofratino	21.0	7.9	1.4	1.0	7.4	−0.5	1.5	2.2

The full model includes all the explanatory variables that were retained after backward elimination in the models accounting for 1. forest structure, 2. abiotic environmental factors and 3. spatial distances. Str:Env|Spa  =  Joint effect of structural and abiotic environmental differences after controlling for the effect of spatial distance. Str:Env:Spa  =  Joint effect of all three types of dissimilarities.

**Figure 3:**
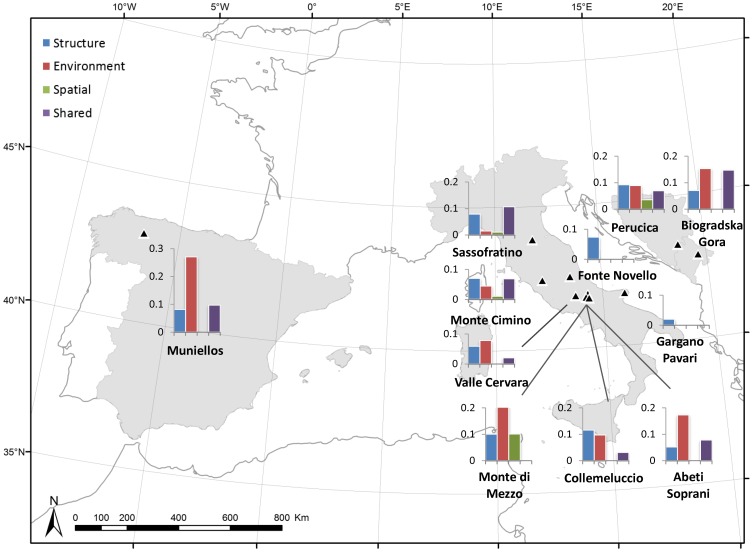
Fractions of Variation explained per stand. Fractions of variance in ground layer species turnover explained by multiple regression on distance matrices models (MRMs) in 11 old-growth beech forest stands. Bar charts report the relative explanatory power of forest structure, abiotic environment and spatial distance. Each pie-chart is placed next to the triangle that indicates the geographical location of the corresponding site. Negative shared fractions are not shown.

The breakdown of variance into fractions explained by the different groups of variables are reported in Table 4 and Fig. 3. Three stands (‘Monte di Mezzo’, ‘Perucica’ and ‘Sassofratino’) returned negative shared fractions, which were very small except in ‘Monte di Mezzo’, where their magnitude was comparable to positive fractions.

Across the 11 stands, we found a significant correlation between explanatory variables' standard deviations (as an estimation of the underlying ecological gradient length) and the amount of variation explained by the corresponding dissimilarity matrices in MRM models in six cases only. These variables were: overstorey richness, canopy density, stone coverage, soil N content, soil silt fraction and water content (Table S3).

### Mixed effect models – Multivariate dispersion

**Figure 4:**
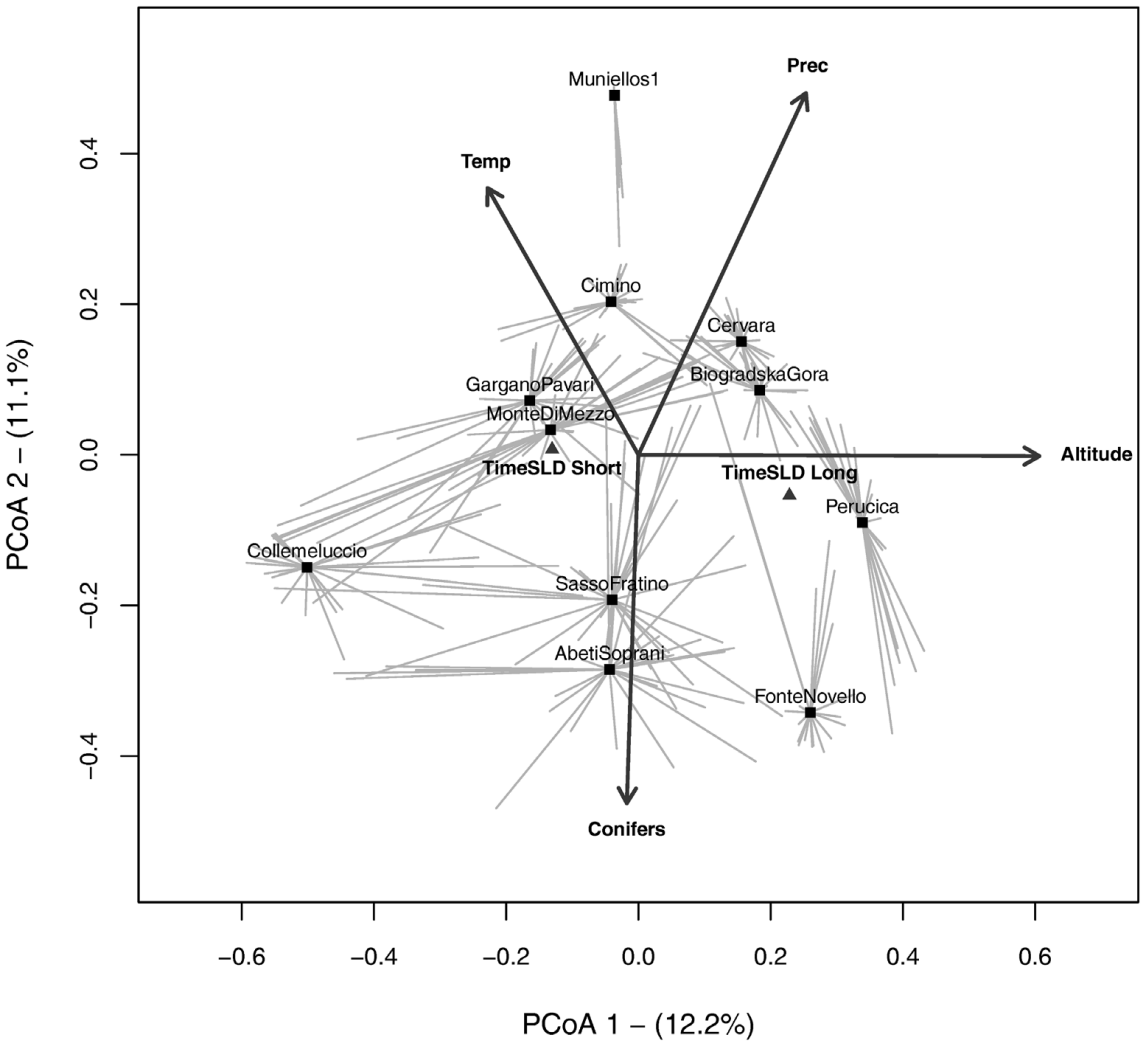
Understorey multivariate dispersion from stand centroids. Multivariate dispersion of quadrats (tips of gray lines) from stand centroids (black squares) in the multivariate space defined by the first two axes of a PCoA calculated on ground layer species composition in 11 old-growth beech forests of southern Europe. Site environmental variables were passively projected on the ordination to help the interpretation of the PCoA axes, either as vectors (quantitative variables) or black triangles (nominal variables). Temp: Average annual mean temperature; Prec: Average annual precipitation; Time SLD  =  Time Since Last Disturbance (Long >100 yrs, Short  =  <100 yrs).

In the PCoA scatterplot based on understorey composition (Fig. 4) the first axis seems to be mainly related to altitude and time since last disturbance, and the second axis to the occurrence of conifers in the overstorey. Mean annual temperature and precipitation were also related to the second PCoA axis. The stands showing the largest dispersion of quadrats around the stand centroid in the multivariate species space (i.e. the highest species turnover between the quadrats and the stand centroid) were ‘Monte Cimino’, ‘Sasso Fratino’ and ‘Gargano Pavari’. Those showing the smallest dispersion were ‘Muniellos’, ‘Fonte Novello’ and ‘Perucica’ (Table S2). With one exception, these were the same sites that showed the highest and lowest beta diversity at q = 2, respectively. Indeed multivariate dispersion was highly correlated with ^2^D_β_ (Pearson's r = 0.94, p<0.001).

Multivariate compositional distance between a quadrat and its stand centroid was positively related to the corresponding differences in stand structure and soil properties (Table 5). The R^2^ of the linear regression between the fitted and the observed values (a rough estimate of the variation explained by the model) was equal to 36.4%.

**Table 5:** Output of the Mixed Effect Model on the response effect of within-stand structural and environmental dissimilarity and spatial distance on ground layer species turnover as modelled by the multivariate distance between quadrats and their stand centroids.

Linear mixed-effects model fit by REML					
Random effects:		Formula: ∼1 | stand			
	Plot	Residual			
StdDev:	0.116	0.167			
					
Fixed effects: Species Turnover ∼ Live structure + Soil					
	Value	Std.Error	DF	t-value	p-value
(Intercept)	0.177	0.048	260	3.691	0.000
Live structure	0.772	0.197	260	3.919	0.000
Soil	0.690	0.216	260	3.199	0.002

A random intercept model with stand as the only random effect was selected as the best-fitting parsimonious model.

## Discussion

### Structural heterogeneity rather than dispersal limitation is the main driver of floristic turnover

Our work provides insights into the mechanisms underlying ground layer species assembly in beech dominated old-growth forests. Environmental filtering, as determined by structural and environmental differences, explained a larger proportion of the variation in species turnover than dispersal limitation, as modelled by spatial distances. Within the stands, species turnover was mostly related to structural heterogeneity and, thereafter, to differences in abiotic environmental variables. The fraction of variation explained by spatial distances was on average very low, and not even statistically significant in most of the stands. Similar results were obtained when the 11 stands were analysed together using mixed effect models. In this case, the only significant predictors of species turnover were structural and soil heterogeneity. However, a large part of the variation in species turnover remained unexplained, which indicates that also other processes may be important, such as neutral dynamics.

When environmental factors are spatially autocorrelated, it is difficult to statistically separate the effects of dispersal limitation and environmental or habitat filtering [5], [21], [43]. A study performed in an old-growth temperate forest in Canada, which avoided this problem by using a sampling design that minimised the spatial autocorrelation of environmental variables, found environmental factors to be more important than dispersal-related mechanisms [6]. In our data, environmental and structural dissimilarities were only weakly or not at all correlated with spatial distances, and very little variance in species turnover was hence jointly explained by geographical and environmental distances (Table 4). The results of the mixed effect models further support the conclusion that dispersal limitation had only a small effect on ground layer species turnover in the southern European temperate forests we studied at the targeted spatial scales. Similar results have been found for tropical forests both at local and regional scales [12], [51], [52].

The low amount of variation explained by the spatial fraction may also be a spurious result due to the uneven number of explanatory variables among sets. Peres-Neto et al. [53] demonstrated for variation partitioning based on raw-data (e.g. RDA or CCA) that the estimation of the variation explained by a set should be corrected for the number of variables included in that set. Since no correction has been developed yet for MRM, we mitigated the effect of having uneven sets of explanatory variables by performing a preliminary selection of significant and non-collinear variables to be retained in each set.

In some stands, MRM returned negative values for the variation shared among sets of variables. This is a well-known problem of all variance partitioning techniques and has been attributed either to suppressor variables (i.e., a regressor having close to zero correlation with the response variable and a correlation with another regressor, which in turn is correlated with the response variables), or to two strongly correlated predictors with strong effects on the dependent variables of opposite signs [53]. However, since we performed both a preliminary and a backward selection of significant variables to be included in the full MRM, and thus minimized multicollinearity, we feel confident on the general validity of our results.

According to the mixed-effect model, within-stand species turnover was driven essentially by the heterogeneity in forest structure and soil variables, with no contribution from other variables such as deadwood, ground layer PAR, topography or within-stand spatial distances. The random intercept model we used supports the interpretation that the slope of the relationship between species turnover and soil or structural dissimilarity remains constant, but the amount of ‘residual’ species turnover between two quadrats with equal structural and soil conditions varies randomly across sites. This ‘residual’ species turnover is likely to include both locally important sources of environmental variability, not significant at the scale of the whole dataset, and other factors related to life-history traits of those species composing the ground layer assemblage. For instance, assemblages dominated by species with limited dispersal capabilities may have different ‘residual’ species turnover than those dominated by wind-dispersed species.

### Length of the ecological gradients and relative roles of different environmental variables

The role of resource-driven niche processes and environmental variation is expected to be more limited at local than at broad spatial scales, because small areas usually harbour shorter ecological gradients than large areas do [10], [54]. On the other hand, the effect of neutral processes, such as dispersal limitation, should be more easily detectable at small than at large geographical distances since it is related to the logarithm of distance [15], [23]. However, the results of our study did not support the expectation that dispersal limitation dominates at local scales [14], [55]. In contrast, structural and environmental heterogeneity always returned a higher amount of variation explained than spatial distances.

When considering our results, one should always keep in mind that the relative importance of different assembly processes critically varies with the spatial scale [9], [16], [55]. In this study, we only considered the within-stand component of ground layer spatial variation (encompassing a distance range of 20–150 m). The small extent (∼1 ha) and grain (25 m^2^) of our study design was aimed at matching the scale at which structural heterogeneity is created and maintained by gap dynamics [56], which is the dominant disturbance regime in southern European temperate old-growth beech forests [57]. Such a spatial scale is too fine to detect variations in the species pools due to biogeographical patterns, but probably too coarse to detect understorey plant autocorrelation occurring within typical neighbourhood sizes for plant populations [6].

The amount of ecological variability sampled in our macroplots varied among stands, also in relation to their developmental stage. Processes that produce horizontal spatial heterogeneity, such as gap development, are active throughout stand development. However, during the old-growth stage these processes increasingly generate within-stand structural heterogeneity [56], [57]. Stands that developed into old-growth conditions a long time ago, are likely to be spatially more heterogeneous than those that only recently attained this condition, with repercussions on other ecological factors such as transmitted light distribution. Forest-floor light availability, as measured both directly (PAR) and using an indirect approach (tree cover, canopy openness) had a significant effect on ground layer species turnover except in four stands: ‘Collemeluccio’, ‘Gargano-Pavari’, ‘Monte di Mezzo’ and ‘Perucica’. The first three of these stands were characterized by a very continuous forest canopy with a scarce occurrence of canopy gaps, and thus a very short forest floor light gradient. Although these forests showed some old-growth features, they were probably still in a developmental stage in which gap-phase dynamics was only just starting, and high levels of horizontal diversification had not yet accumulated. ‘Perucica’ on the other hand, was a well preserved old-growth stand in which several gaps broke the horizontal continuity of the forest canopy, thanks to a relatively long period without human intervention. This was the only stand where ground layer species turnover was significantly related to basal area, probably in relation to its high within-stand variability. The relationships between basal area and ground layer species turnover are indirect, and are not limited to competition for light, but also for above- and below-ground growing space, given that basal area is a proxy for biomass. The relevance of structural variables, such as basal area and deadwood density, and the lack of a significant effect of light for this stand, support the hypothesis that the asymmetric competition exerted by the overstorey on ground layer flora is not only related to the shading effect [4].

Tree-layer composition was a significant predictor of species turnover only in two stands, both dominated by a mixture of broadleaved trees and conifers. It seems likely that, in those stands where the canopy composition was dominated by a single species (‘Fonte Novello’ and ‘Valle Cervara’), compositional variation became uninformative. For the other stands, the compositional gradient may still be too short to be relevant, suggesting that overstorey species turnover may be more effective in predicting ground-layer species turnover at broader spatial scales. Indeed, when longer environmental gradients are considered, all components of the vegetation are likely to react to the environmental variation and hence become more strongly correlated [16], [51], [58].

The effect of top-soil heterogeneity on ground layer turnover was related either to soil carbon and nitrogen concentration or to soil texture. Soil C and N concentration were especially important in mixed stands, likely in relation to patchy accumulation of beech and fir litter, whose different nutrient contents and decomposition rates probably create a fine scale mosaic of heterogeneous topsoil parameters. Interestingly, in our study we did not observe any significant effect of topsoil pH variability on species turnover. In a study on the effect of fine-scale soil variability on plant distribution in German beech forests, very little variation was explained by pH alone, suggesting that the effect of pH may not have a substantial effect on ground layer species turnover at this scale [59].

Although we only recorded disturbance as ordinal indices, difference in disturbance level was a significant explanatory variable of species turnover in the stand ‘Monte di Mezzo’ which is a 300 ha forest patch heavily affected by the foraging activity of the wild boar. As in other natural reserves in central Italy, the population of wild boar has exploded in the recent decades, causing high pressure on ground-layer flora that accounts for most of the wild boar diet. Rooting activity is thus locally an important source of spatial variation in the ground-layer, both through direct herbivory and through its effect on soil features.

Our results partly supported the hypothesis that the relative importance of different environmental and structural variables depends on their degree of fine-scale heterogeneity. Only six variables showed a direct relationship between their standard deviation (as a measure of gradient length) and the fraction of variation explained by their dissimilarity matrices in MRM models (Table S3). However, all of these represent ecological factors generally thought to directly influence ground layer flora, namely light availability (canopy openness), soil water availability and nitrogen content, as well as competition by overstorey species. This is in agreement with the notion that the power of a statistical test to recognise an existing causal link between dependent and explanatory variables depends on the degree of heterogeneity in the explanatory variable. Although this result lends support to the interpretation that the observed relationships are indeed causal, our study was observational and not manipulative and may thus be vulnerable to potential (unknown) confounding factors.

Confounding factors may include unmeasured environmental variables, as well as the choice of inappropriate response models. These are usually identified as potential reasons accounting for the amount of ‘unexplained variation’ when performing variation partitioning [60]. In our study we tried to take into account as many sources of environmental heterogeneity that potentially affect ground-layer species turnover as possible. The stands in which MRM returned the highest amount of ‘unexplained variation’ were two monospecific beech stands (‘Fonte Novello’, ‘Gargano-Pavari’), with a relatively uniform forest structure (continuous canopy cover with a low proportion of gaps) and little topographical heterogeneity (Table S1 and S2). Given the relatively homogeneous forest conditions, we expected these stands to return a beta-diversity value lower than average, but this was true only for ‘Fonte Novello’ (Table 3). In these stands some unmeasured abiotic factors, such as phosphorus or microelement concentrations in the soil, or depth of the water table, might have been particularly important. Unmeasured forms of natural disturbance (e.g. browsing by ungulates) might also be locally important in creating floristic patterns. We do not think, instead, that the amount of unexplained variation was related to lack-of-fit of the response model: given the local scale and the relatively short length of most environmental gradients within our study sites, the linear model underlying MRM appears justified (see also Figure S1).

We conclude that ground-layer species turnover strongly depends on niche-related processes, such as environmental filtering, which depend primarily on forest structural and environmental heterogeneity. Since old-growth forests represent only a very small percentage of forests in southern Europe, the need of developing and implementing silvicultural practices aimed at restoring old-growth structural attributes in regrowth and secondary forests is now widely recognized [25]. Based on the significant effects of structural heterogeneity on species turnover variation, our results suggest that silvicultural approaches that mimic the amount and patterns of structural heterogeneity observed in reference old-growth forests may have a positive effect on the conservation and restoration of high levels of ground layer species turnover in managed forests. However, conservation needs to pay attention also to the identity (life-history traits, conservation status) of the species to be conserved and not just aim at maximizing local heterogeneity [71]. Only with a complementary approach that includes the preservation of strict forest reserves, to ensure long-term ecological continuity of some reference forest stands, on the one hand, and a close-to-nature management of production forests on the other, we will be able to match both conservation goals and other social and economic needs.

## Acknowledgments

We would like to thank Borja Jiménez-Alfaro, Emanuela Carli, Carmen Giancola, Eleonora Giarrizzo, Giorgia Martina, Manuel Palma, Danijela Stešević and Antonio Zoccola for help during the fieldwork, and Monica Zanini for the analysis of soil samples. We are grateful to Renzo Motta and Matteo Garbarino for logistic support. Further logistic assistance was provided by State Forestry Corps, ‘Abruzzo, Lazio and Molise National Park’, ‘Gran Sasso and Monti della Laga National Park’, ‘ASBUC Intermesoli’, ‘Giardino della Flora Appenninica di Capracotta’, ‘Jardín Botánico Atlántico’ (Gijón, Spain). The manuscript was partly prepared during FMS's stay at University of Turku.

## Supporting Information

Figure S1 **Relationships between understorey dissimilarities and a subset of environmental dissimilarities.**
(DOCX)

Appendix S1 **An R script to perform true diversity decomposition.**
(TXT)

Table S1 **Averages and standard deviations of environmental variables.**
(DOCX)

Table S2 **Stand average multivariate dispersion from group centroid.**
(DOCX)

Table S3 **Correlation analysis between environmental and structural variables' standard deviations, and their marginal effect on ground layer species turnover.**
(DOCX)

## References

[1] Watt AS (1947) Pattern and process in the plant community. The Journal of Ecology 35: 1–22.

[2] Tuomisto H, Ruokolainen K, Yli-Halla M (2003) Dispersal, environment, and floristic variation of western Amazonian forests. Science 299: 241–244. 12522248 10.1126/science.1078037

[3] Burrascano S, Anzellotti I, Carli E, Del Vico E, Facioni L, et al (2013) Drivers of beta-diversity variation in Bromus erectus semi-natural dry grasslands. Appl Veg Sci 16: 404–416.

[4] Burton JI, Mladenoff DJ, Clayton MK, Forrester JA (2011) The roles of environmental filtering and colonization in the fine-scale spatial patterning of ground-layer plant communities in north temperate deciduous forests. J Ecol 99: 764–776.

[5] Dray S, Pélissier R, Couteron P, Fortin MJ, Legendre P, et al (2012) Community ecology in the age of multivariate multiscale spatial analysis. Ecology 82: 257–275.

[6] Gilbert B, Lechowicz MJ (2004) Neutrality, Niches, and Dispersal in a Temperate Forest Understory. Proc Natl Acad Sci U S A 101: 7651–7656. 15128948 10.1073/pnas.0400814101 PMC419661

[7] Laliberte E, Paquette A, Legendre P, Bouchard A (2009) Assessing the scale-specific importance of niches and other spatial processes on beta diversity: a case study from a temperate forest. Oecologia 159: 377–388. 19018575 10.1007/s00442-008-1214-8

[8] Cottenie K (2005) Integrating environmental and spatial processes in ecological community dynamics. Ecol Lett 8: 1175–1182. 21352441 10.1111/j.1461-0248.2005.00820.x

[9] Myers JA, Chase JM, Jiménez I, Jørgensen PM, Araujo-Murakami A, et al (2013) Beta-diversity in temperate and tropical forests reflects dissimilar mechanisms of community assembly. Ecol Lett 16: 151–157. 23113954 10.1111/ele.12021

[10] Ruokolainen K, Linna A, Tuomisto H (1997) Use of Melastomataceae and pteridophytes for revealing phytogeographical patterns in Amazonian rain forests. J Trop Ecol 13: 243–256.

[11] Peet R (1978) Forest vegetation of the Colorado Front Range: Patterns of species diversity. Vegetatio 37: 65–78.

[12] Jones MM, Tuomisto H, Clark DB, Olivas P (2006) Effects of mesoscale environmental heterogeneity and dispersal limitation on floristic variation in rain forest ferns. J Ecol 94: 181–195.

[13] Karst J, Gilbert B, Lechowicz MJ (2005) Fern community assembly: the roles of chance and the environment at local and intermediate scales. Ecology 86: 2473–2486.

[14] Gravel D, Canham CD, Beaudet M, Messier C (2006) Reconciling niche and neutrality: the continuum hypothesis. Ecol Lett 9: 399–409. 16623725 10.1111/j.1461-0248.2006.00884.x

[15] Hubbell SP (2001) The Unified Neutral Theory of Biodiversity and Biogeography. Princeton, New Jersey, USA: Princeton University Press. 375 p.

[16] Jones MM, Ferrier S, Condit R, Manion G, Aguilar S, et al (2013) Strong congruence in tree and fern community turnover in response to soils and climate in central Panama. J Ecol 101: 506–516.

[17] Winter S, Moller GC (2008) Microhabitats in lowland beech forests as monitoring tool for nature conservation. For Ecol Manag 255: 1251–1261.

[18] Burrascano S, Rosati L, Blasi C (2009) Plant species diversity in Mediterranean old-growth forests: A case study from central Italy. Plant Biosyst 143: 190–200.

[19] Saitta, Bernicchia A, Gorjon SP, Altobelli E, Granito VM, et al (2012) Biodiversity of wood-decay fungi in Italy. Plant Biosyst 146: 488–488.

[20] Norden B, Appelqvist T (2001) Conceptual problems of Ecological Continuity and its bioindicators. Biodivers Conserv 10: 779–791.

[21] Tuomisto H, Ruokolainen K (2006) Analyzing or explaining beta diversity? Understanding the targets of different methods of analysis. Ecology 87: 2697–2708. 17168014 10.1890/0012-9658(2006)87[2697:aoebdu]2.0.co;2

[22] López-Martínez JO, Hernández-Stefanoni JL, Dupuy JM, Meave JA (2013) Partitioning the variation of woody plant β-diversity in a landscape of secondary tropical dry forests across spatial scales. J Veg Sci 24: 33–45.

[23] Nekola JC, White PS (1999) The distance decay of similarity in biogeography and ecology. J Biogeogr 26: 867–878.

[24] Burrascano S, Keeton WS, Sabatini FM, Blasi C (2013) Commonality and Variability in the Structural Attributes of Moist Temperate Old-growth Forests: a Global Review. For Ecol Manag 291: 458–479.

[25] Bauhus J, Puettmann K, Messier C (2009) Silviculture for old-growth attributes. For Ecol Manag 258: 525–537.

[26] Capotorti G, Zavattero L, Anzellotti I, Burrascano S, Frondoni R, et al (2012) Do National Parks play an active role in conserving the natural capital of Italy? Plant Biosyst 146: 258–265.

[27] Gilliam FS (2007) The ecological significance of the herbaceous layer in temperate forest ecosystems. Bioscience 57: 845–858.

[28] Larsen JB (1995) Ecological stability of forests and sustainable silviculture. For Ecol Manag 73: 85–96.

[29] Royo AA, Carson WP (2006) On the formation of dense understory layers in forests worldwide: consequences and implications for forest dynamics, biodiversity, and succession. Can J For Res 36: 1345–1362.

[30] Rivas-Martinez S, Rivas-Saenz S (2009) Worldwide Bioclimatic Classification System, 1996–2009. Spain, http://www.globalbioclimatics.org: Phytosociological Research Center. Accessed 2013 Mar 1.

[31] Calamini G, Maltoni A, Travaglini D, Iovino F, Nicolaci A, et al (2011) Stand structure attributes in potential old-growth forests in the Apennines, Italy. Italia Forestale e Montana 66: 365–381.

[32] Motta R, Bjelanovic I, Borgogno Mondino E, Curovic M, Garbarino M, et al. (*in press*) Analysis of the spatio-temporal dynamics of mixed beechsilver fir-Norway spruce old-growth forests of Biogradska Gora (Montenegro) and Perucica (Bosnia and Herzegovina). Plant Biosyst.

[33] Aguirre O, Hui GY, von Gadow K, Jimenez J (2003) An analysis of spatial forest structure using neighbourhood-based variables. For Ecol Manag 183: 137–145.

[34] Hunter ML (1990) Wildlife, forests and forestry: principles of managing forests for biological diversity. Englewood Cliffs: Prentice Hall.

[35] McCune B, Keon D (2002) Equations for potential annual direct incident radiation and heat load. J Veg Sci 13: 603–606.

[36] Tuomisto H (2010) A diversity of beta diversities: straightening up a concept gone awry. Part 2. Quantifying beta diversity and related phenomena. Ecography 33: 23–45.

[37] Tuomisto H (2010) A diversity of beta diversities: straightening up a concept gone awry. Part 1. Defining beta diversity as a function of alpha and gamma diversity. Ecography 33: 2–22.

[38] Tuomisto H (2011) Commentary: do we have a consistent terminology for species diversity? Yes, if we choose to use it. Oecologia 167: 903–911. 10.1007/s00442-011-2124-8 21947497

[39] Tuomisto H (2010) A consistent terminology for quantifying species diversity? Yes, it does exist. Oecologia 164: 853–860. 20978798 10.1007/s00442-010-1812-0

[40] Hill MO (1973) Diversity and Evenness: A Unifying Notation and Its Consequences. Ecology 54: 427–432.

[41] Jost L (2006) Entropy and diversity. Oikos 113: 363–375.

[42] Jost L (2007) Partitioning diversity into independent alpha and beta components. Ecology 88: 2427–2439. 18027744 10.1890/06-1736.1

[43] Legendre P, Borcard D, Peres-Neto PR (2005) Analyzing beta diversity: Partitioning the spatial variation of community composition data. Ecol Monogr 75: 435–450.

[44] Lichstein JW (2007) Multiple regression on distance matrices: a multivariate spatial analysis tool. Plant Ecol 188: 117–131.

[45] Duivenvoorden JF, Svenning JC, Wright SJ (2002) Ecology - Beta diversity in tropical forests. Science 295: 636–637. 11809957 10.1126/science.295.5555.636

[46] Legendre P, Lapointe FJ, Casgrain P (1994) Modeling brain evolution from behavior - A permutational regression approach. Evolution 48: 1487–1499. 28568410 10.1111/j.1558-5646.1994.tb02191.x

[47] Whittaker J (1984) Model Interpretation from the Additive Elements of the Likelihood Function. Journal of the Royal Statistical Society Series C (Applied Statistics) 33: 52–64.

[48] Anderson MJ (2006) Distance-Based Tests for Homogeneity of Multivariate Dispersions. Biometrics 62: 245–253. 16542252 10.1111/j.1541-0420.2005.00440.x

[49] Zuur AF, Ieno EN, Walker NJ, Saveliev AA, Smith GM (2009) Mixed Effect Models and Extensions in Ecology with R. Berlin: Springer-Verlag.

[50] R Development Core Team (2011) R: A language and environment for statistical computing Vienna, Austria: R Foundation for Statistical Computing.

[51] Ruokolainen K, Tuomisto H, Macia MJ, Higgins MA, Yli-Halla M (2007) Are floristic and edaphic patterns in Amazonian rain forests congruent for trees, pteridophytes and Melastomataceae? J Trop Ecol 23: 13–25.

[52] Poulsen AD, Tuomisto H, Balslev H (2006) Edaphic and Floristic Variation within a 1-ha Plot of Lowland Amazonian Rain Forest1. Biotropica 38: 468–478.

[53] Peres-Neto PR, Legendre P, Dray S, Borcard D (2006) Variation partitioning of species data matrices: Estimation and comparison of fractions. Ecology 87: 2614–2625. 17089669 10.1890/0012-9658(2006)87[2614:vposdm]2.0.co;2

[54] MacArthur RH, Wilson EO (1967) The theory of island biogeography. Princeton, NJ: Princeton University Press.

[55] Guèze M, Paneque-Gálvez J, Luz AC, Pino J, Orta-Martínez M, et al (2013) Determinants of tree species turnover in a southern Amazonian rain forest. J Veg Sci 24: 284–295.

[56] Nagel TA, Svoboda M (2008) Gap disturbance regime in an old-growth Fagus-Abies forest in the Dinaric Mountains, Bosnia-Herzegovina. Can J For Res 38: 2728–2737.

[57] Rugani T, Diaci J, Hladnik D (2013) Gap Dynamics and Structure of Two Old-Growth Beech Forest Remnants in Slovenia. PLoS ONE 8: e52641. 23308115 10.1371/journal.pone.0052641 PMC3538742

[58] Burrascano S, Sabatini FM, Blasi C (2011) Testing indicators of sustainable forest management on understorey composition and diversity in southern Italy through variation partitioning. Plant Ecol 212: 829–841.

[59] Bruelheide H, Udelhoven P (2005) Correspondence of the fine-scale spatial variation in soil chemistry and the herb layer vegetation in beech forests. For Ecol Manag 210: 205–223.

[60] Borcard D, Legendre P, Drapeau P (1992) Partialling out the Spatial Component of Ecological Variation. Ecology 73: 1045–1055.

[61] Lombardi F, Chirici G, Marchetti M, Tognetti R, Lasserre B, et al (2010) Deadwood in forest stands close to old-growthness under Mediterranean conditions in the Italian Peninsula. Italia Forestale e Montana 65: 481–504.

[62] Čurović M, Stešević D, Medarević M, Cvjetićanin R, Pantić D, et al (2011) Ecological and structural characteristics of monodominant montane beech forests in the national park Biogradska Gora, Montenegro. Arch Biol Sci 63: 429–440.

[63] Burrascano S, Lombardi F, Marchetti M (2008) Old-growth forest structure and deadwood: Are they indicators of plant species composition? A case study from central Italy. Plant Biosyst 142: 313–323.

[64] Piovesan G, Di Filippo A, Alessandrini A, Biondi F, Schirone B (2005) Structure, dynamics and dendroecology of an old-growth Fagus forest in the Apennines. J Veg Sci 16: 13–28.

[65] Sabatini FM, Burrascano S, Blasi C (2010) Niche heterogeneity and old-growth conservation value. Italia Forestale e Montana 65: 621–636.

[66] Sabatini FM, Jiménez-Alfaro B, Burrascano S, Blasi C, (*in press*) Drivers of herb-layer species diversity in two unmanaged temperate forests in northern Spain. Community Ecol.

[67] Fernández Prieto JA, Bueno Sánchez A (1992) A New Classification of the Forests of the Muniellos Biological Reserve in Northwest Spain. Vegetatio 102: 33–46.

[68] Nagel TA, Svoboda M, Rugani T, Diaci J (2010) Gap regeneration and replacement patterns in an old-growth Fagus-Abies forest of Bosnia-Herzegovina. Plant Ecol 208: 307–318.

[69] Bianchi L, Bottacci A, Calamini G, Maltoni A, Mariotti B, et al (2011) Structure and dynamics of a beech forest in a fully protected area in the northern Apennines (Sasso Fratino, Italy). iForest - Biogeosciences and Forestry 4: 136–144.

[70] Travaglini D, Paffetti D, Bianchi L, Bottacci A, Bottalico F, et al (2012) Characterization, structure and genetic dating of an old-growth beech-fir forest in the northern Apennines (Italy). Plant Biosyst 146: 175–188.

[71] Sabatini FM, Burton JI, Scheller RM, Amatangelo KL, Mladenoff DJ (*in press*) Functional diversity of ground-layer plant communities in old-growth and managed northern hardwood forests. Appl Veg Sci. doi: 10.1111/avsc.12083

