# Supplementary material for: Ground Layer Plant Species Turnover and Beta Diversity in Southern-European Old-Growth Forests
Source: PLoS One. 2014 Apr 18;9(4):e95244. doi: 10.1371/journal.pone.0095244 (PMC3991708; doi:10.1371/journal.pone.0095244)
Supplement: Figure S1 — Relationships between understorey dissimilarities and a subset of environmental dissimilarities. (DOCX) [file pone.0095244.s001.docx]

Figure S5 – Distribution of understorey dissimilarities (histograms, first column) and relationships between understorey dissimilarities (y axis) and a subset of environmental dissimilarities
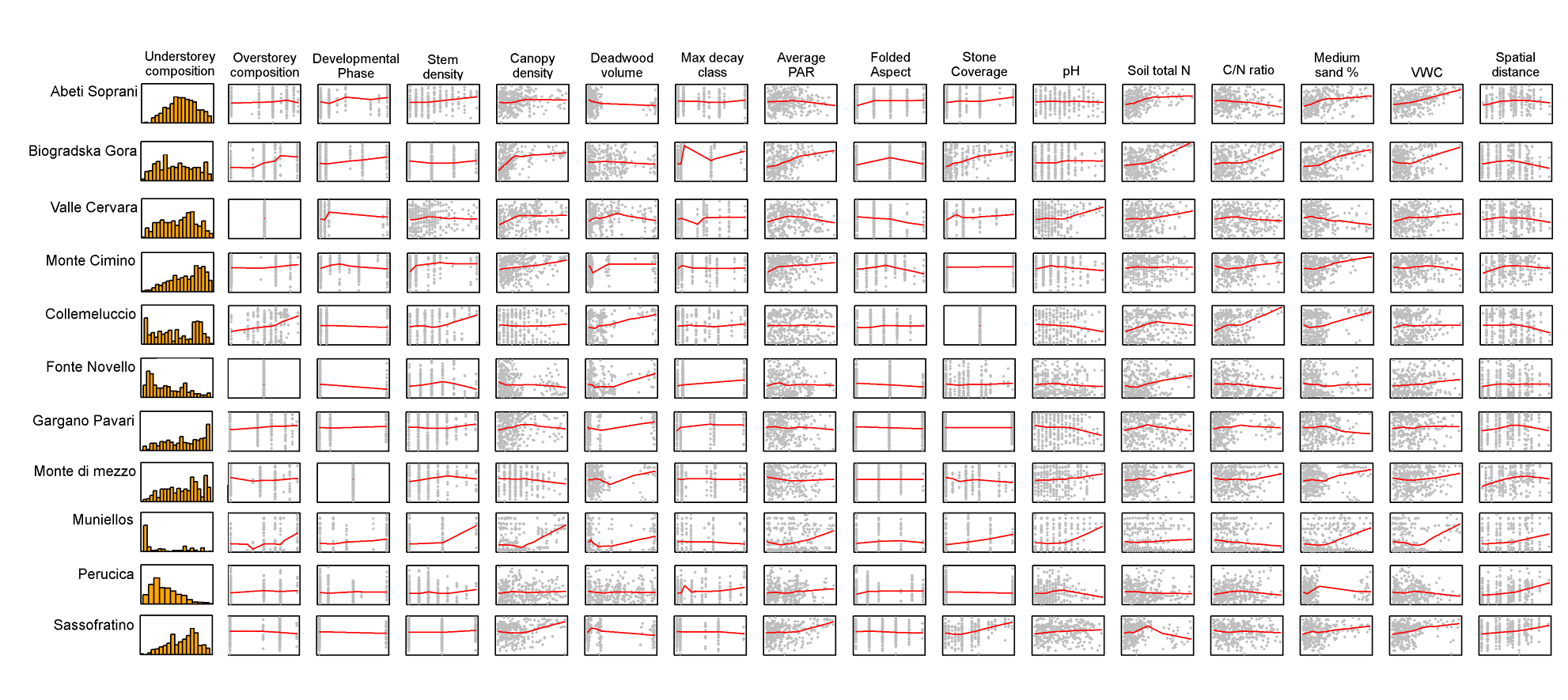
selected from Fig. 1 (x axis) in 11 old-growth beech stands in Southern Europe. Red lines represent LOWESS (locally weighted scatterplot smoothing) regressions.
