# Supplementary material for: Ground Layer Plant Species Turnover and Beta Diversity in Southern-European Old-Growth Forests
Source: PLoS One. 2014 Apr 18;9(4):e95244. doi: 10.1371/journal.pone.0095244 (PMC3991708; doi:10.1371/journal.pone.0095244)
Supplement: Table S1 — Averages and standard deviations of environmental variables. (DOCX) [file pone.0095244.s003.docx]

Table S2 – Values of environmental variables in each of 11 old-growth forest stands. Average ± standard deviation are given for continuous variables, median and interquartile distance (in parentheses) for ordinal variables, and mode for nominal variables. Topographic position: v – slope, s – summit. Slope position: c – upper slope, l – middle slope, v – lower slope. Texture: AL – silty clay, FL – silt loam, L – silty, FLA – silty clay Loam, F – loamy, FS – sandy loam. Developmental stage: 1 – gap, 2 – stand initiation (dominant trees with dbh <2.5 cm), 3 – stand exclusion (dominant trees with dbh <20 cm), 4 – early biostatic (dominant trees with 20<dbh<40 cm), 5 – late biostatic (dominant trees with dbh > 40 cm).

| **Subset** | **Variable name** | **Abeti Soprani** | **Biogradska Gora** | **Cervara** | **Cimino** | **Collemeluccio** | **Fonte Novello** | **Gargano- Pavari** | **Monte di Mezzo** | **Muniellos** | **Perucica** | **Sasso Fratino** |
| --- | --- | --- | --- | --- | --- | --- | --- | --- | --- | --- | --- | --- |
| 1a | Tree richness | 2.12 ± 0.83 | 1.56 ± 0.77 | 1 ± 0 | 1.2 ± 0.5 | 3.36 ± 0.91 | 1 ± 0 | 2.28 ± 0.61 | 2.2 ± 0.65 | 1.76 ± 0.83 | 1.92 ± 0.49 | 1.64 ± 0.57 |
| 1b | Tree cover | 78.8 ± 15.36 | 67 ± 19.9 | 62.4 ± 23.68 | 75.8 ± 32.46 | 86.8 ± 9 | 86.8 ± 8.02 | 85 ± 13.62 | 94.6 ± 6.44 | 63 ± 20.92 | 70.8 ± 11.87 | 89.2 ± 11.87 |
|  | Shrub cover | 9.04 ± 9.13 | 3.36 ± 5.93 | 25.32 ± 19.02 | 22.4 ± 29.05 | 10.16 ± 15.47 | 1.04 ± 3.1 | 9.8 ± 5.1 | 1.8 ± 2.33 | 0.08 ± 0.4 | 1.36 ± 1.75 | 0.08 ± 0.28 |
|  | Develop. phase | 5(1) | 5(1) | 3(2) | 5(2) | 4(1) | 5(0) | 5(2) | 5(0) | 5(4) | 5(1) | 5(0) |
|  | Basal Area (Prism) | 49.77 ± 12.18 | 34.16 ± 7.88 | 31.68 ± 9.74 | 41.51 ± 8.89 | 47.66 ± 7.45 | 50.69 ± 13 | 38.38 ± 5.64 | 40.59 ± 6.1 | 32.78 ± 8.42 | 52.43 ± 11.12 | 47.2 ± 5.78 |
|  | Stem density | 3.04 ± 2.98 | 1.12 ± 0.97 | 6.36 ± 5.77 | 1.24 ± 2.47 | 2.72 ± 2.19 | 1.44 ± 1.61 | 2.48 ± 1.98 | 1.4 ± 1.8 | 0.44 ± 0.58 | 1.92 ± 1.63 | 0.44 ± 0.71 |
|  | Basal area (quadrat) | 0.19 ± 0.21 | 0.23 ± 0.36 | 0.07 ± 0.12 | 0.13 ± 0.22 | 0.11 ± 0.12 | 0.25 ± 0.36 | 0.13 ± 0.19 | 0.1 ± 0.16 | 0.07 ± 0.14 | 0.37 ± 0.45 | 0.14 ± 0.26 |
|  | Canopy opennes | 3.66 ± 1.89 | 11.47 ± 9.13 | 7.97 ± 5.44 | 8.48 ± 6.37 | 2.67 ± 0.96 | 2.44 ± 2.35 | 5.09 ± 3.69 | 3.13 ± 0.74 | 19.09 ± 18.17 | 10.68 ± 5.58 | 4.85 ± 2.09 |
|  | Uniform Angle Index | 0.42 ± 0.19 | 0.51 ± 0.2 | 0.53 ± 0.18 | 0.5 ± 0.19 | 0.47 ± 0.22 | 0.6 ± 0.24 | 0.44 ± 0.17 | 0.53 ± 0.17 | 0.48 ± 0.12 | 0.43 ± 0.17 | 0.42 ± 0.17 |
|  | Sp. Mingling Index | 0.15 ± 0.26 | 0.69 ± 0.32 | 0 ± 0 | 0.12 ± 0.25 | 0.2 ± 0.3 | 0 ± 0 | 0.16 ± 0.29 | 0.53 ± 0.32 | 0.41 ± 0.41 | 0.35 ± 0.3 | 0.37 ± 0.35 |
|  | DBHDM Index | 0.52 ± 0.37 | 0.41 ± 0.42 | 0.56 ± 0.33 | 0.53 ± 0.38 | 0.37 ± 0.39 | 0.52 ± 0.43 | 0.33 ± 0.3 | 0.64 ± 0.43 | 0.36 ± 0.38 | 0.33 ± 0.37 | 0.47 ± 0.42 |
|  | Distance closest tree (> 40 cm dbh) | 3.52 ± 2.38 | 4.39 ± 2.86 | 5.58 ± 2.48 | 3.83 ± 1.96 | 3.36 ± 1.77 | 3 ± 1.93 | 4.49 ± 2.42 | 4.16 ± 1.91 | 5.31 ± 2.63 | 3.7 ± 1.89 | 4.48 ± 2.23 |
| 1c | Dw volume | 0.25 ± 0.72 | 2.78 ± 3.91 | 0.58 ± 1 | 0.25 ± 0.76 | 0.06 ± 0.12 | 0.41 ± 0.77 | 0.6 ± 2.11 | 0.07 ± 0.08 | 1.05 ± 2.64 | 4.41 ± 5.13 | 1.55 ± 3.91 |
|  | Dw density | 1.36 ± 1.68 | 4.12 ± 2.77 | 3.96 ± 3.16 | 2.48 ± 1.94 | 2.12 ± 2.4 | 5.44 ± 2.96 | 5.04 ± 3.4 | 4.04 ± 2.44 | 1.24 ± 1.45 | 4.76 ± 3.22 | 4.08 ± 2.64 |
|  | Max decay class | 2(4) | 4(1) | 3(1) | 3(2) | 3(4) | 4(1) | 4(1) | 4(2) | 3(4) | 4(1) | 4(1) |
|  | num. decay classes | 1 ± 1.22 | 2.24 ± 1.05 | 1.76 ± 0.97 | 1.32 ± 0.8 | 1.32 ± 1.18 | 2.4 ± 0.71 | 2.4 ± 1.15 | 2.2 ± 1.04 | 0.88 ± 0.83 | 2.48 ± 1.29 | 2.08 ± 0.95 |
| 1d | Morning PAR | 2.07 ± 1.09 | 3.5 ± 0.8 | 3.35 ± 1.14 | 1.39 ± 1.37 | 2.23 ± 1.09 | 2.69 ± 1.11 | 2.62 ± 1.1 | 1.37 ± 0.3 | 4.42 ± 1.32 | 3.61 ± 1.07 | 1.82 ± 0.89 |
|  | Noon PAR | 3.17 ± 1.17 | 3.44 ± 0.58 | 3.7 ± 1.48 | 2.02 ± 1.42 | 2.86 ± 1.11 | 3.38 ± 1 | 2.67 ± 1.09 | 2.09 ± 0.67 | 4.73 ± 1.51 | 4.61 ± 1.06 | 2.4 ± 1.14 |
|  | Afternoon PAR | 2.14 ± 1.06 | 3.42 ± 0.6 | 2.46 ± 0.88 | 2.63 ± 1.38 | 2.62 ± 1.08 | 2.35 ± 0.84 | 2.36 ± 1.49 | 1.8 ± 0.57 | 3.87 ± 1 | 3.45 ± 0.46 | 0.96 ± 0.56 |
|  | Average PAR | 2.93 ± 0.91 | 3.48 ± 0.63 | 3.58 ± 1.11 | 2.52 ± 1.32 | 2.92 ± 0.9 | 3.13 ± 0.85 | 2.92 ± 1.08 | 1.89 ± 0.45 | 4.59 ± 1.18 | 4.24 ± 0.84 | 2.07 ± 0.98 |
| 2a | Slope | 9.08 ± 9.4 | 24.78 ± 7.04 | 30.6 ± 8.82 | 3.6 ± 3.82 | 5.12 ± 5.26 | 13.98 ± 2.78 | 10.26 ± 3.29 | 18.8 ± 12.93 | 24.04 ± 8.79 | 18 ± 5.69 | 19.14 ± 4.85 |
|  | Folded aspect | 37.8 ± 31.63 | 13.5 ± 18.37 | 26.1 ± 22.2 | 110.45 ± 46.53 | 50.62 ± 30.59 | 68.4 ± 16.53 | 17.1 ± 16.28 | 34.2 ± 17.33 | 18 ± 18.37 | 24.3 ± 36.12 | 100.8 ± 33.82 |
|  | Pot. solar irradiation | -0.36 ± 0.1 | -0.84 ± 0.21 | -0.87 ± 0.32 | -0.28 ± 0.07 | -0.33 ± 0.09 | -0.33 ± 0.08 | -0.47 ± 0.08 | -0.58 ± 0.28 | -0.77 ± 0.17 | -0.65 ± 0.21 | -0.23 ± 0.18 |
|  | Topographic position | v | v | v | s | v | v | v | v | v | v | v |
|  | Slope position | v | l | l | c | v | l | l | l | c | l | v |
|  | Rock coverage | 1.12 ± 2.37 | 9.92 ± 17.38 | 9.6 ± 10.2 | 3.96 ± 7.47 | 0.2 ± 0.58 | 3.36 ± 3.05 | 0 ± 0 | 9.44 ± 8.67 | 2.8 ± 10.32 | 0.64 ± 0.86 | 26.56 ± 16.29 |
|  | Stone coverage | 1.44 ± 2.58 | 10.2 ± 14.2 | 5.88 ± 3.9 | 0.16 ± 0.55 | 0 ± 0 | 4.8 ± 3.95 | 1.4 ± 2.29 | 4.48 ± 2.66 | 4.2 ± 4.49 | 0.24 ± 0.44 | 6.92 ± 6.04 |
| 2b | Soil pH | 5.96 ± 0.42 | 4.43 ± 0.25 | 6.19 ± 0.46 | 4.77 ± 0.2 | 7.01 ± 0.53 | 6.18 ± 0.82 | 5.8 ± 0.48 | 5.68 ± 0.62 | 3.75 ± 0.3 | 4.91 ± 0.51 | 6.28 ± 1.13 |
|  | Soil Organic Matter | 9.47 ± 3.18 | 10.77 ± 3.8 | 13.21 ± 3.2 | 7.32 ± 1.83 | 8.27 ± 2.74 | 5.04 ± 2.64 | 9.06 ± 1.95 | 7.76 ± 2.89 | 5.34 ± 1.9 | 12.22 ± 3.24 | 6.54 ± 3.31 |
|  | Soil tot. N | 0.46 ± 0.13 | 0.63 ± 0.23 | 0.95 ± 0.19 | 0.45 ± 0.07 | 0.45 ± 0.12 | 0.3 ± 0.11 | 0.53 ± 0.13 | 0.47 ± 0.13 | 0.25 ± 0.07 | 0.49 ± 0.08 | 0.32 ± 0.16 |
|  | C/N ratio | 11.55 ± 1.65 | 10.26 ± 1.99 | 8.15 ± 1.71 | 9.35 ± 1.94 | 10.67 ± 1.89 | 9.98 ± 2.48 | 9.98 ± 1.52 | 9.6 ± 1.3 | 12.62 ± 3.48 | 14.56 ± 3.17 | 11.64 ± 1.59 |
|  | Soil stone content | 1(2) | 1(1) | 1(2) | 0(0) | 0(0) | 0(1) | 1(1) | 0(0) | 2(1) | 0(1) | 0(1) |
|  | Coarse Sand % | 6.65 ± 5.85 | 12.38 ± 5.97 | 1.42 ± 0.93 | 13.76 ± 4.36 | 2.12 ± 1.97 | 3.42 ± 3.02 | 7.47 ± 1.86 | 1.95 ± 1.15 | 19.54 ± 9.49 | 2.02 ± 1.26 | 4.3 ± 3.15 |
|  | Medium Sand % | 2.28 ± 1.42 | 5.59 ± 2.26 | 0.92 ± 0.59 | 8.17 ± 2.24 | 1 ± 0.72 | 9.59 ± 3.41 | 4.29 ± 0.92 | 1.71 ± 0.66 | 7.55 ± 2.59 | 6.18 ± 3.53 | 10.18 ± 4.65 |
|  | Fine Sand % | 3.66 ± 1.99 | 7.77 ± 4.06 | 2.08 ± 0.54 | 10.74 ± 2.83 | 5.08 ± 1.74 | 26.9 ± 4.43 | 8.29 ± 1.29 | 6.95 ± 1.66 | 23.35 ± 5.06 | 10.42 ± 2.98 | 30.29 ± 6.09 |
|  | Silt % | 45.78 ± 11.87 | 58.17 ± 13.25 | 87.38 ± 4.26 | 50.66 ± 10.53 | 53.94 ± 6.48 | 45.24 ± 9.52 | 75.77 ± 4.49 | 67.14 ± 7.49 | 44.32 ± 14.61 | 72.3 ± 6.5 | 44.03 ± 9.33 |
|  | Clay % | 41.64 ± 15.44 | 16.06 ± 4.19 | 8.2 ± 4.68 | 16.69 ± 5.91 | 37.85 ± 8.35 | 15.28 ± 5.08 | 4.16 ± 3.36 | 22.25 ± 8.24 | 5.25 ± 2.17 | 9.09 ± 6.17 | 11.21 ± 4.59 |
|  | Texture | AL | FL | L | FL | FLA | F | FL | FL | FS | FL | F |
|  | VWC | 36.55 ± 14.54 | 29.54 ± 5.79 | 11.07 ± 2.5 | 20.27 ± 3.45 | 37.48 ± 8.65 | 23.22 ± 2.72 | 16.41 ± 2.62 | 25.34 ± 5.46 | 14.33 ± 2.5 | 30.81 ± 3.88 | 13.46 ± 4.33 |
|  | Litter cover | 72 ± 16.07 | 80.8 ± 12.88 | 83.2 ± 9.88 | 89.6 ± 5.58 | 61.6 ± 23.57 | 79.2 ± 11.15 | 92.4 ± 6.94 | 92.8 ± 5.97 | 39.4 ± 25.34 | 86 ± 6.45 | 46.4 ± 14.4 |
|  | Litter depth | 1.72 ± 0.66 | 1.64 ± 0.67 | 2.44 ± 0.51 | 5.24 ± 5.46 | 1.68 ± 0.8 | 2.28 ± 0.9 | 4.16 ± 1.59 | 3.34 ± 0.87 | 5.48 ± 1.57 | 1.9 ± 0.46 | 3.7 ± 2.13 |
| 2c | Disturbance-Trampling | 1(2) | 0(0) | 0(0) | 2(1) | 0(2) | 0(0) | 0(0) | 2(3) | 0(0) | 0(0) | 0(0) |
|  | Disturbance-Rooting | 0(0) | 0(0) | 0(0) | 0(0) | 0(0) | 0(0) | 0(0) | 0(0) | 0(0) | 0(0) | 0(0) |
|  | Disturbance-Timber harvest | 0(0) | 0(0) | 0(0) | 0(0) | 0(0) | 0(0) | 0(0) | 0(0) | 0(0) | 0(0) | 0(0) |
|  | Disturbance-Water Erosion | 0(0) | 0(0) | 0(0) | 0(0) | 0(0) | 0(0) | 0(0) | 0(0) | 0(0) | 0(0) | 0(2) |
