# Supplementary material for: Ground Layer Plant Species Turnover and Beta Diversity in Southern-European Old-Growth Forests
Source: PLoS One. 2014 Apr 18;9(4):e95244. doi: 10.1371/journal.pone.0095244 (PMC3991708; doi:10.1371/journal.pone.0095244)
Supplement: Table S2 — Stand average multivariate dispersion from group centroid. (DOCX) [file pone.0095244.s004.docx]

Table S3 – Average multivariate dispersion from group centroid for each stand, in the multivariate space determined by understorey species composition, and other subsets of structural and environmental variables. For each subset of explanatory variables (see table 2) we calculated a compound dissimilarity matrix, that we used to calculate the multivariate distance of each quadrat to its centroid in the corresponding multivariate PCoA space.

|  | Ground layer | Overstorey | Forest structure | Deadwood | Floor PAR | Topography | Soil | Disturbance |
| --- | --- | --- | --- | --- | --- | --- | --- | --- |
|  |  | 1a | 1b | 1c | 1d | 2a | 2b | 2c |
| Abeti Soprani | 0.411 | 0.364 | 0.134 | 0.122 | 0.050 | 0.149 | 0.142 | 0.121 |
| Biogradska Gora | 0.361 | 0.366 | 0.143 | 0.145 | 0.024 | 0.089 | 0.132 | 0.000 |
| Valle Cervara | 0.341 | 0.000 | 0.135 | 0.137 | 0.037 | 0.086 | 0.078 | 0.000 |
| Monte Cimino | 0.457 | 0.087 | 0.157 | 0.109 | 0.031 | 0.116 | 0.110 | 0.023 |
| Collemeluccio | 0.325 | 0.384 | 0.112 | 0.141 | 0.033 | 0.110 | 0.109 | 0.070 |
| Fonte Novello | 0.204 | 0.000 | 0.102 | 0.089 | 0.020 | 0.105 | 0.099 | 0.038 |
| Gargano-Pavari | 0.449 | 0.230 | 0.113 | 0.130 | 0.023 | 0.107 | 0.076 | 0.010 |
| Monte di Mezzo | 0.426 | 0.153 | 0.102 | 0.124 | 0.016 | 0.079 | 0.083 | 0.117 |
| Muniellos | 0.160 | 0.391 | 0.158 | 0.135 | 0.103 | 0.089 | 0.122 | 0.000 |
| Perucica | 0.155 | 0.107 | 0.132 | 0.188 | 0.046 | 0.062 | 0.079 | 0.000 |
| Sasso Fratino | 0.457 | 0.213 | 0.115 | 0.125 | 0.017 | 0.181 | 0.138 | 0.073 |
