# Supplementary material for: Ground Layer Plant Species Turnover and Beta Diversity in Southern-European Old-Growth Forests
Source: PLoS One. 2014 Apr 18;9(4):e95244. doi: 10.1371/journal.pone.0095244 (PMC3991708; doi:10.1371/journal.pone.0095244)
Supplement: Table S3 — Correlation analysis between environmental and structural variables' standard deviations, and their marginal effect on ground layer species turnover. (DOCX) [file pone.0095244.s005.docx]

Table S4 – Correlation analysis between explanatory variables’ standard deviations, and the marginal effect of the corresponding dissimilarity matrix used as explanatory variable in MRM models on ground layer species turnover. **Bold** – significant at p < 0.05, *italic* – significant at p <0.1

|  | Pearson's ρ | p-value |
| --- | --- | --- |
| Overstorey richness | 0.62 | **0.044** |
| Tree cover | 0.23 | 0.504 |
| Shrub cover | -0.06 | 0.869 |
| Develop. phase | 0.03 | 0.930 |
| Basal Area (Prism) | 0.22 | 0.510 |
| Stem density | -0.08 | 0.811 |
| Basal area (quadrat) | -0.33 | 0.320 |
| Canopy openness | 0.71 | **0.014** |
| Uniform Angle Index | 0.12 | 0.730 |
| Sp. Mingling Index | 0.31 | 0.348 |
| DBHDM Index | 0.19 | 0.585 |
| Distance closest large live tree | -0.32 | 0.345 |
| Deadwood volume | -0.48 | 0.135 |
| Deadwood density | -0.02 | 0.959 |
| Max decay class | -0.57 | *0.064* |
| num. decay classes | 0.43 | 0.190 |
| Volumetric Water Content | 0.74 | **0.009** |
| Morning PAR | -0.09 | 0.789 |
| Noon PAR | -0.10 | 0.773 |
| Afternoon PAR | -0.42 | 0.196 |
| Average PAR | -0.22 | 0.521 |
| Slope | 0.46 | 0.152 |
| Folded aspect | -0.21 | 0.540 |
| Pot. solar irradiation | 0.22 | 0.520 |
| Rock coverage | 0.28 | 0.411 |
| Stone coverage | 0.89 | **0.000** |
| Soil pH | 0.00 | 0.992 |
| Soil Organic Matter | 0.57 | *0.065* |
| Soil tot. N | 0.61 | **0.047** |
| C/N ratio | -0.01 | 0.979 |
| Coarse Sand % | 0.30 | 0.365 |
| Medium Sand % | -0.08 | 0.808 |
| Fine Sand % | 0.43 | 0.184 |
| Silt % | 0.65 | **0.031** |
| Clay % | 0.59 | *0.055* |
| Litter cover | -0.01 | 0.985 |
| Litter depth | 0.55 | *0.082* |
| Soil stone content | 0.33 | 0.317 |
